# Interactions of Carbon Dioxide and Food Odours in *Drosophila*: Olfactory Hedonics and Sensory Neuron Properties

**DOI:** 10.1371/journal.pone.0056361

**Published:** 2013-02-15

**Authors:** Cécile P. Faucher, Monika Hilker, Marien de Bruyne

**Affiliations:** 1 Institute of Biology - Neurobiology, Freie Universität Berlin, Berlin, Germany; 2 Institute of Biology - Applied Zoology, Freie Universität Berlin, Berlin, Germany; INRA-UPMC, France

## Abstract

Behavioural responses of animals to volatiles in their environment are generally dependent on context. Most natural odours are mixtures of components that can each induce different behaviours when presented on their own. We have investigated how a complex of two olfactory stimuli is evaluated by *Drosophila* flies in a free-flying two-trap choice assay and how these stimuli are encoded in olfactory receptor neurons. We first observed that volatiles from apple cider vinegar attracted flies while carbon dioxide (CO_2_) was avoided, confirming their inherent positive and negative values. In contradiction with previous results obtained from walking flies in a four-field olfactometer, in the present assay the addition of CO_2_ to vinegar increased rather than decreased the attractiveness of vinegar. This effect was female-specific even though males and females responded similarly to CO_2_ and vinegar on their own. To test whether the female-specific behavioural response to the mixture correlated with a sexual dimorphism at the peripheral level we recorded from olfactory receptor neurons stimulated with vinegar, CO_2_ and their combination. Responses to vinegar were obtained from three neuron classes, two of them housed with the CO_2_-responsive neuron in ab1 sensilla. Sensitivity of these neurons to both CO_2_ and vinegar *per se* did not differ between males and females and responses from female neurons did not change when CO_2_ and vinegar were presented simultaneously. We also found that CO_2_-sensitive neurons are particularly well adapted to respond rapidly to small concentration changes irrespective of background CO_2_ levels. The ability to encode temporal properties of stimulations differs considerably between CO_2_- and vinegar-sensitive neurons. These properties may have important implications for in-flight navigation when rapid responses to fragmented odour plumes are crucial to locate odour sources. However, the flies’ sex-specific response to the CO_2_-vinegar combination and the context-dependent hedonics most likely originate from central rather than peripheral processing.

## Introduction

Chemical stimuli are among the most basic environmental cues that guide animals to food and mates or away from toxins and predators. The apparently inherent hedonic value of certain odours to certain animals has led to the labelling of volatiles as “attractants” and “repellents” [1]. However, behavioural responses to environmental stimuli are generally flexible and a given stimulus can elicit different responses depending on time of day, physiological state, previous experience or gender. The responses by blood-sucking bugs to olfactory cues depend on endogenous rhythms as well as on exogenous cues [2] and can switch from attraction to repellency [3]. Behavioural decisions may also depend on experimental design. For instance, while the role of CO_2_ in mosquito feeding behaviour is now well understood, initial investigation produced conflicting results that depended on experimental conditions [4]. In addition, most natural olfactory stimuli are mixtures of odorants, and behavioural responses often are not consistent with simple additive effects of the components [5], [6], [7]. Modifications of neural signals leading to complex responses to mixtures may take place at different levels of olfactory processing [8]. For instance, recent evidence suggests that inhibition caused by one odorant at the level of a single sensory neuron can modify the response to another odorant either by reducing it or by modifying temporal firing properties [9], [10].

How are olfactory systems adapted to the behavioural ecology of animals? How does their design determine what odours are avoided and what odours are attractive? To address these questions, we need first to study how complex olfactory stimuli are evaluated and how their negative and positive values depend on context. The robust hedonic values of odour from apple cider vinegar and carbon dioxide (CO_2_) in the behaviour of the fly *Drosophila melanogaster* are particularly well suited for such an investigation. Apple cider vinegar, a product of fruit fermentation, is attractive to *Drosophila* in a range of assays [11], [12], [13]. Its attractiveness is likely due to volatile components that also occur in more natural stimuli such as fruit. By contrast, CO_2_ has been shown to elicit avoidance behaviour both in a T-maze [14] and in a four-field olfactometer [15]. While the attractiveness of vinegar appears logical for an animal that feeds on fermenting fruit like *Drosophila,* it is not immediately obvious why flies avoid carbon dioxide. CO_2_ is an omnipresent stimulus, found at approximately 0.03% in ambient air. Changes in CO_2_ concentration depend on ventilation and emissions from metabolic activity by animals, plants and microorganisms, and are relevant signals for many insects [16], [17]. In most cases the CO_2_ signal has a positive value, enhanced by the simultaneous detection of host odours, as in haematophagous insects such as mosquitoes [18] and in phytophagous insects such as moths [19], [20]. *Drosophila* is attracted to yeast on over-ripe and fermenting fruits [9], [21]–[23], which are used for feeding, oviposition and as a mating site. Both decaying fruit and yeast produce CO_2_, so a positive hedonic value would be expected, particularly when combined with host-specific odorants. The negative hedonic value of CO_2_ may be because it is the active component of the escape inducing “*Drosophila* stress odorant” [14]. However, even though stressed flies increase their CO_2_ output [14], [15], many other organisms may do the same, and emissions from ripening fruit are generally higher [15].

In *Drosophila,* CO_2_ is processed by a specific neuronal pathway. It is detected by a single class of specifically tuned olfactory receptor neurons (ORNs) on the antennae, the ab1C neurons [14], [24]. These neurons differ from most ORNs because their response is mediated by two members of the gustatory receptor family (Gr21a and Gr63b, [25], [26]) whereas most other ORNs express a member of the odorant receptor (Or) family co-expressed with the Orco receptor [26]–[28]. The CO_2_-specific neuronal pathway further differs by the ab1C neurons’ target in the antennal lobe, the V-glomerulus, which lacks contra-lateral innervations [29] and whose projection neurons follow a different trajectory to higher brain regions [30]. The non-olfactory activation of ab1C neurons has been shown to induce the typical avoidance behaviour, demonstrating the specificity of this neuronal pathway [31].

What happens when an olfactory stimulus with an inherently positive value (vinegar) is combined with a negative stimulus (CO_2_) that is processed by a distinct olfactory pathway? We previously studied the interaction between CO_2_ and vinegar in the four-field olfactometer and found that vinegar enhanced the behavioural sensitivity of female flies to CO_2_, making them avoid a concentration that was not avoided on its own [15]. In this assay, flies were in a relatively small enclosed space, only able to walk in and out of discrete homogenous odour fields while their behaviour was observed for a short period of 10 min. These results confirmed the negative hedonic value of CO_2_.

In the present study we wondered whether the interaction between CO_2_ and vinegar would be different when *Drosophila* would be free-flying and would have more time to orient themselves to more natural odour plumes. We used a novel two-choice trap assay and verified the positive and negative hedonic value of vinegar and CO_2_, respectively. However, in contradiction with our previous observations, we found that the combination of CO_2_ and vinegar was more attractive than vinegar alone. This combination effect was still observed only in females. To investigate whether the female-specific modulation of the behavioural response was due to different properties of male and female olfactory receptor neurons, we determined how the odours of CO_2_, vinegar and their combination are encoded by neurons from large basiconic sensilla, which house the CO_2_-selective neurons. We found that the ORN sensitivity to CO_2_ and vinegar *per se* is similar for males and females, supporting the behavioural observations in response the individual stimuli. We also demonstrated that the responses of female ab1C neurons to CO_2_ are not modified by the simultaneous stimulation with vinegar suggesting that the female-specific response to the mixture is not caused by effects in the periphery. Finally, we show considerable differences between CO_2_- and vinegar-sensitive neurons in their ability to encode the temporal properties of odour stimulations.

## Results

### CO_2_ increases Vinegar Attractiveness in a Free-flight Two-trap Cage Assay

**Figure 1:**
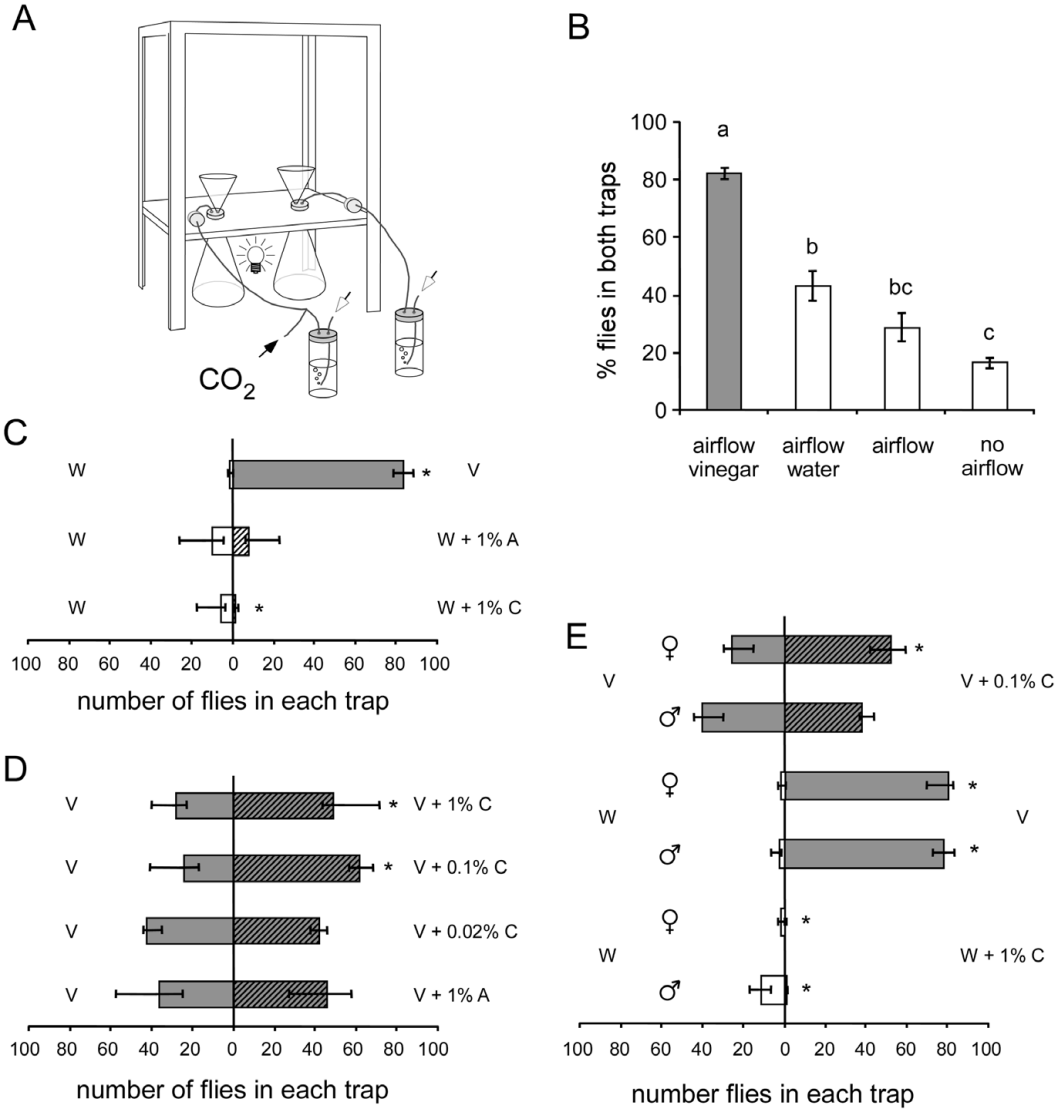
Behavioural responses of flies to vinegar and CO_2_. A. Schematic drawing of the two-trap cage assay used to assess *Drosophila* olfactory behaviour. A controlled airflow (white arrows) delivered through two bubble vials entered traps in a 30×30×30 cm cage. The traps consisted of a glass flask closed with a silicon plug and a plastic funnel by which the odour is released and flies can enter. About 100 flies (males and females 50∶50) were tested for 5 hours in darkness except for a small light below the opaque floor of the cage. A small controlled flow of CO_2_ or pressurised air could be added to one of the traps (black arrow). **B.** No choice assays. Percentage of flies trapped in the two flasks when both either contained 3 ml apple cider vinegar, 3 ml distilled water, were empty or empty without airflow. Different letters above the bars denote significant differences (Kruskal-Wallis test, p<0.05; post-hoc Mann–Whitney U-test with Bonferroni correction, p<0.05). Values are medians over 10 replicates and error bars indicate the 25% and 75% percentiles. **C, D, E.** Two-choice assays. The numbers of flies trapped in the control trap are indicated on the left, those in the test trap on the right. Adding the two bars indicates the total number of flies trapped. **C.** Numbers of flies trapped in two-choice situations between water (W) and either apple cider vinegar (V), water with 1% pressurised air (A) or 1% CO_2_ (C). **D.** Numbers of flies trapped in two-choice situations between apple cider vinegar alone (V) and vinegar mixed with either 1% pressurized air (A) or different concentrations of CO_2_ (C). **E.** Percentage of flies trapped when males and females were tested separately (100 males or 100 females). Asterisks in C, D and E indicate a significant difference between the number of flies caught in the two traps (Friedman-ANOVA, p<0.05; followed by Wilcoxon signed rank test with sequential Bonferroni correction, p<0.05).

We investigated the behavioural responses of *Drosophila* to CO_2_, odour from apple cider vinegar, and their combinations in a free-flight trap-based assay. Flies, released in a cage, were given a choice between two funnels, each emitting an odour-laden airstream (Figures 1A, S1). We first tested whether flies were attracted to the traps when the two funnels presented identical stimuli. When no airflow and no odour was applied, the small light source below the cage weakly attracted flies to the funnels (Figure 1B), setting a baseline of attraction from which both increase and decrease could be quantified. The catch efficiency increased with the emission of a humidified air stream, and adding odour of apple cider vinegar made the traps strongly attractive, with more than 80% of flies caught in the two flasks (Figure 1B). Conversely, distilled water was significantly less attractive than vinegar (Figure 1B). For all conditions tested, there was no bias toward either of the traps (Wilcoxon matched-pairs test, p>0.05).

We then offered a choice of two different traps to the flies. Tested against distilled water, the vinegar-baited trap was clearly preferred, and the overall catch remained very high (80%, Figure 1C). When testing two traps with distilled water, adding pressurised air (containing ambient levels of CO_2_) to one of them had no effect on the fly distribution, while the total catch was still around 40%. However, adding 1% CO_2_ to one of the traps resulted in a significant reduction in the number of flies entering that trap and also lowered the total catch to around 15% (Figure 1C). We conclude that the basic tendency of *Drosophila* flies to be attracted to vinegar and repelled by CO_2_ can be observed under these free-flying conditions. When comparing the avoidance of 1% CO_2_ to our previous results in the four-field olfactometer and in a classical T-maze assay, the response indices show similar levels of CO_2_ avoidance (Figure S2).

We then asked how adding CO_2_ to vinegar would affect its attractiveness in this new assay. In contrast to our previous olfactometer bioassays, flies were more attracted to a combination of CO_2_ and vinegar than to vinegar alone (Figure 1D). This preference was dependent on CO_2_ concentration: attraction to vinegar plus 1% and 0.1% CO_2_ was significantly stronger than to vinegar alone, while a concentration of 0.02% CO_2_ failed to elicit any significant change in the attraction to vinegar. The addition of pressurised air to one trap did not affect the flies’ attraction to the vinegar odour. Thus, CO_2_ modified the attraction to vinegar in both the cage and olfactometer assays, but in apparently opposite directions.

We then wondered whether the effect of CO_2_ on the attractiveness of vinegar in the cage assay is also sex-specific as it was in the olfactometer. The comparison of the number of males and the number of females caught in the traps baited with CO_2_ and vinegar revealed no significant difference (Wilcoxon matched-pairs tests, p>0.05, not shown). However, when males and females were tested separately, females significantly preferred the CO_2_-vinegar combination over vinegar alone, whereas males did not (Figure 1E). Interestingly, when vinegar and CO_2_ were tested separately, we found that males and females were both attracted to the vinegar-baited traps, and both avoided the CO_2_-baited traps (Figure 1E). It is possible that some aspect of the flieś response to CO_2_ was sex-specific because the total catch in both traps was lowered more in females than in males (not shown, Mann-Whitney U-test, p = 0.001). These results confirm our earlier olfactometer results on the similar responses of males and females to CO_2_ and vinegar when presented on their own, as well as on the female-specific orientation behaviour to their combination, but contradict the direction of that orientation. While CO_2_ in a vinegar background was strongly avoided by females in the four-field olfactometer, it increased vinegar attractiveness in the two-trap assay.

### Vinegar-sensitive Neurons are Found in Large Basiconic Sensilla

**Figure 2:**
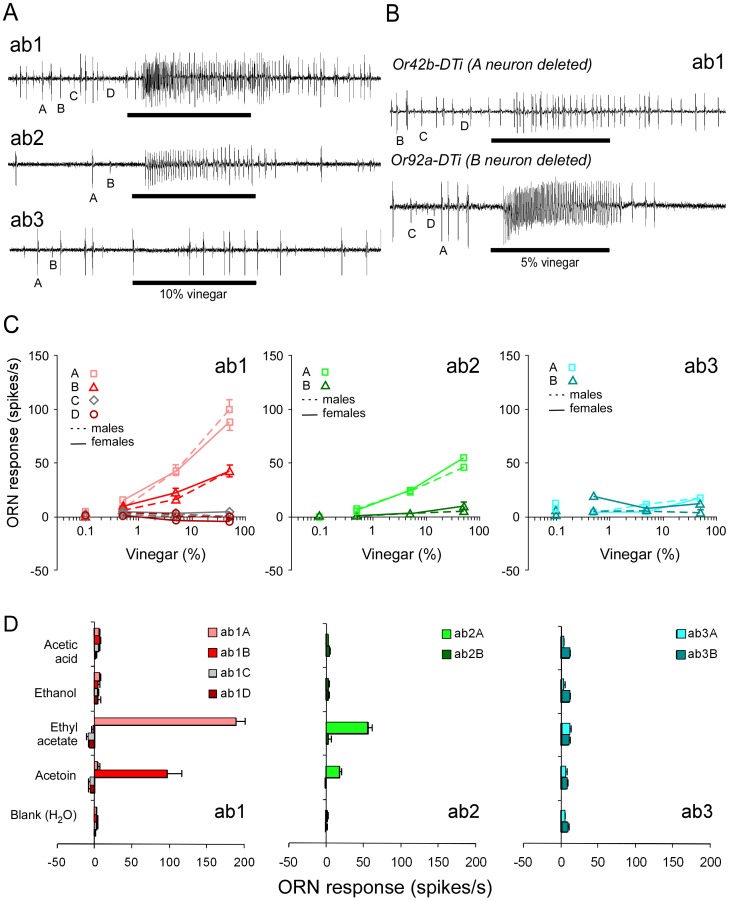
Responses from male and female receptor neurons to vinegar and its main components. A. Representative traces of extracellular recordings from ab1, ab2, and ab3 sensilla in response to the odour of apple cider vinegar (10% dilution in distilled water). Action potentials fired by specific neurons are identified as A, B, C or D. Stimulus duration (500 ms) is indicated by the black bar. **B.** Deletion of either A or B neurons in ab1 sensilla confirms that both ab1A and ab1B neurons responded to apple cider vinegar with ab1A dominating. A Diphtheria toxin transgene (UAS-DTi) was expressed by using the Or42b-Gal4 (A) or Or92a-Gal4 (B) driver to ablate neurons. **C.** Responses from neurons in ab1, ab2, and ab3 sensilla of male and female flies to a range of vinegar dilutions (in distilled water) applied to filter paper. Values are means ± SEM. Most SEM are too small to be seen (N_males_ = 3–11, N_females_ = 4–9). **D.** Responses to the main components of apple cider vinegar diluted in distilled water at concentrations equivalent to 50% vinegar. Values are means ± SEM (N = 8–19).

To investigate the neuronal basis of the female-specific response to CO_2_ when perceived with vinegar odour, we recorded the responses of antennal olfactory receptor neurons (ORNs) to odour from apple cider vinegar and its most abundant components. We recorded the responses from 8 different classes of ORN in the three large basiconic sensilla distinguished by their odour-specific responses and different action potential amplitudes [24]. These three sensilla contain most of the neurons that innervate the glomeruli shown to respond to vinegar [11]. Vinegar odour elicited clear responses from neurons in ab1 and ab2 sensilla, and no significant responses from the two neurons in ab3 sensilla (Figure 2A). The identity of the neurons responding to vinegar odour in the ab1 sensillum was confirmed by ablating either the A or B neuron. We used targeted expression of the Diphtheria toxin via the Gal4-UAS system, employing either Or42b-GAL4 or Or92a-GAL4 constructs, to drive expression in ab1A or B respectively. Ablation of ab1A neurons left only a minor response from the undamaged ab1B neurons to the odour of apple cider vinegar (Figure 2B). By contrast ab1A neurons showed robust responses to vinegar when ab1B neurons were ablated, indicating that this neuron is largely responsible for the vinegar-induced response in ab1 sensilla.

The dose-response relations show that ab1A is most sensitive to vinegar odours, followed by ab1B and ab2A (Figure 2C). We did not observe any difference in sensitivity between males and females in these three neuron classes at any given dose (Mann-Whitney U-test, p>0.05). Next, we asked which components of the odour of apple cider vinegar are most likely to excite these ORNs. We tested the four most abundant volatiles found in the headspace of commercial apple cider vinegars at concentrations comparable to that in 5% vinegar [32]. The response spectra for ab1, ab2, and ab3 neurons are given in Figure 2D. The main component of vinegar odour, acetic acid, did not excite any of these neurons, neither did ethanol. By contrast, ethyl acetate strongly excited ab1A and ab2A neurons, while acetoin stimulated ab1B and, to a lesser extent, ab2A. These results show that among large basiconic sensilla neuronal responses to apple cider vinegar are strongest in ab1 sensilla and do not differ between the sexes. In addition, the responses to vinegar odour are most likely due to its ethyl acetate and acetoin content. In the following sections we focused our attention on ab1 sensilla because they house the CO_2_-specific ORNs as well as the two neurons most sensitive to ethyl acetate and acetoin. We hypothesised that this may be the site for interaction between CO_2_ and vinegar.

### Coding Properties of CO_2_-sensitive ab1C Neurons in Males and Females

**Figure 3:**
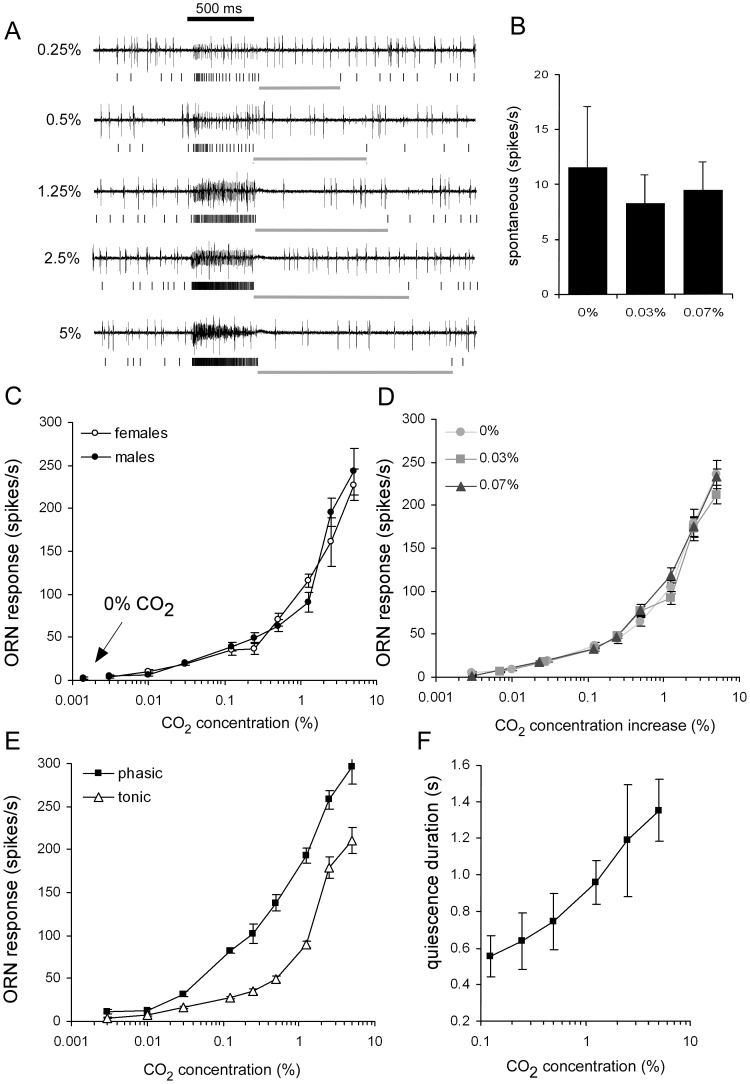
Response properties of the ab1C neurons in males and females to CO_2_. A. Representative traces of extracellular recordings from an ab1 sensillum showing excitatory responses of the ab1C neuron to increasing CO_2_ doses. Stimulus duration (500 ms) is indicated by the black bar. Vertical lines below each trace identify C neuron action potentials, and the quiescence period following stimulation is indicated by a grey line. **B.** CO_2_ background concentration did not affect spontaneous firing (N = 17–23, Kruskal-Wallis test, p>0.05). **C.** Responses from ab1C neurons to 500 ms pulses of a wide range of CO_2_ concentrations (N_males_ = 6–12; N_females_ = 7–9). **D.** Responses to CO_2_ concentrations in various backgrounds: CO_2_-free air (0%; same data as in D, sexes grouped), ambient levels (0.03%, N = 13–18) and CO_2_-enriched air (0.07%, N = 15–17). **E.** Dynamic encoding of CO_2_ concentrations as the ab1C neuron firing rates for the phasic (initial 100 ms) and tonic (final 400 ms) responses differed. (N = 13–19). **F.** The duration of post-stimulus quiescence as indicated in A was dose-dependent. (N = 9–13). All values are means ± SEM.

Next, we examined the physiological properties of CO_2_-sensitive neurons. The ab1C neurons fire spikes with amplitudes well below that of ab1A and B while clearly larger than those of ab1D. The recording traces in Figure 3A show the increasing firing rate of such a neuron with increasing concentrations of CO_2_. These excitatory responses closely matched the duration of the stimulus, with a phasic burst of activity, followed by a lower more tonic frequency and quiescence immediately after stimulation when firing dropped well below the spontaneous activity recorded before the stimulation. Because our behavioural experiments (this paper and [15]) were carried out with room air (∼0.07% CO_2_) and electrophysiological recordings are usually done using synthetic air without CO_2_, we measured the spontaneous activity of ab1C neurons in the absence of CO_2_, in ambient air (∼0.03%) and in room air conditions. Interestingly, their spontaneous activity was not significantly different after exposure to the three background concentrations (Figure 3B). We then investigated the sensitivity of male and female ab1C neurons to a range of increasing CO_2_ concentrations in these three background conditions. First, in the absence of a CO_2_ background, female neurons did not respond significantly different from male neurons as indicated by nearly overlapping dose-response curves (Figure 3C). The curves also show a response of 20 spikes/s to 0.03% CO_2_, a concentration corresponding to ambient levels, demonstrating that both males and females can detect CO_2_ at this level with similar accuracy. We then measured the same dose-response relationships in backgrounds of 0.03% and 0.07% CO_2_. We found that the continuous presence of CO_2_ did not interfere with ab1C sensitivity to changes in CO_2_ levels (Figure 3D). Regardless of the CO_2_ background concentration, the neurons could detect a 0.03% increase even if that background was more than twice the increment tested (0.07%). As in the absence of CO_2_ background, we did not see any difference between males and females (not shown), and data were grouped for clarity. Incidentally, a small drop in CO_2_ concentration below the background actually reduced firing by ab1C (Figure S3).

As shown above, ab1C neurons typically responded in a phasic-tonic pattern, with the initial sharp rise in firing frequency providing a phasic onset of the response and the post-stimulus quiescence an offset. A comparison of the firing rates for the first 100 ms to the firing rates during the last 400 ms clearly reveals their different dose-dependencies (Figure 3E). Phasic responses increased almost linearly with log-step concentrations, while tonic responses were more sigmoidal, resulting in higher sensitivity during the initial 100 ms of stimulation. The response to stimulus offset, *i.e.* the duration of the quiescence period after stimulation, also appears to be related to concentration in a near linear way (Figure 3F). As a result, these neurons are extremely sensitive to small fluctuations of CO_2_ concentration around ambient levels and particularly good at encoding stimulus on- and offset. We did not observe differences between the sexes.

### No Evidence for Direct or Indirect Interaction between CO_2_ and Vinegar in ab1 Neurons

**Figure 4:**
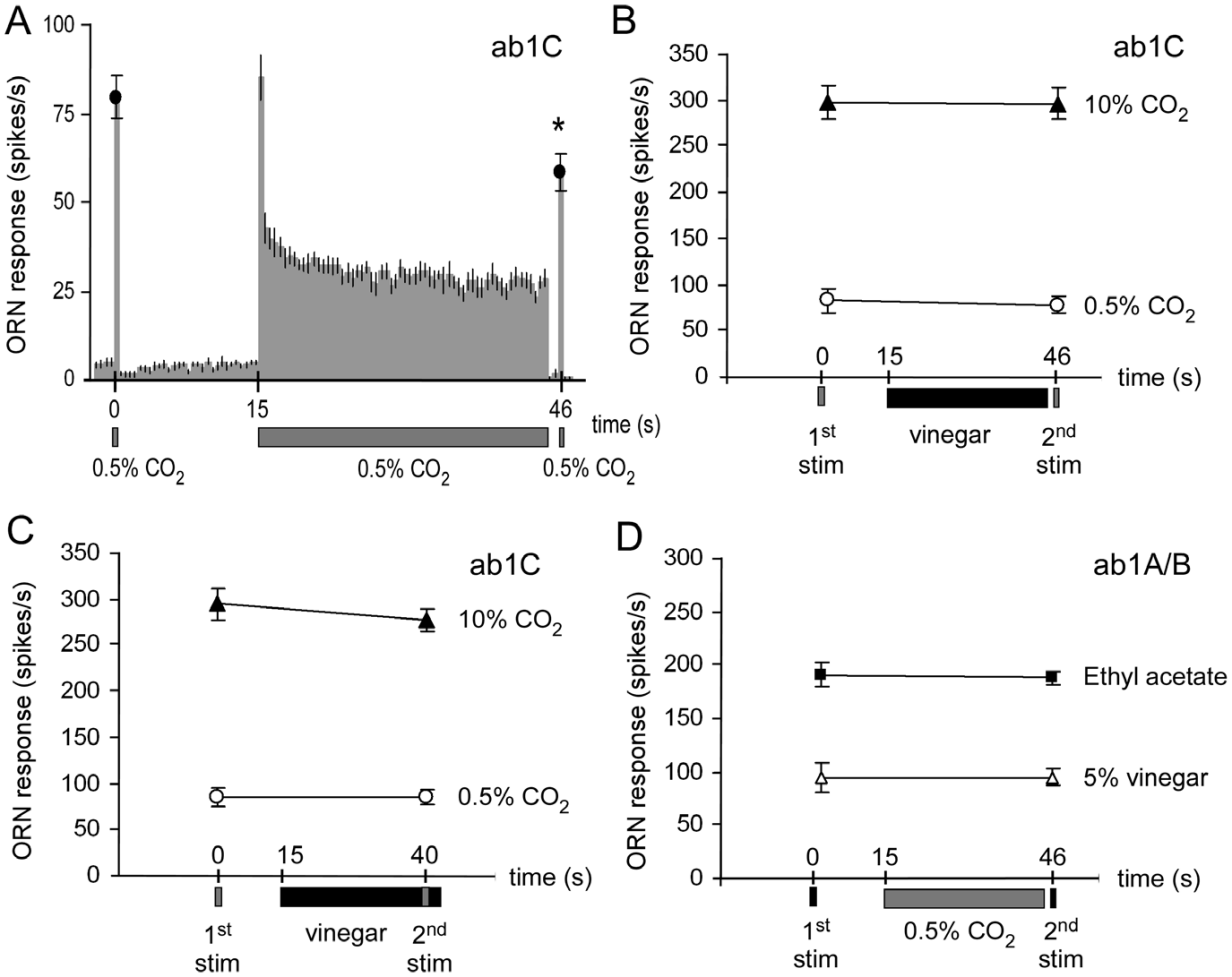
Female ab1 neurons are not affected by the activity of their neighbouring neurons. A. Adaptation can be observed in the spiking activity (500 ms bins) of ab1C neurons before, during, and after prolonged exposure to 0.5% CO_2_. Stimulation was as follows: a first 500 ms pulse followed by a 15 sec rest, a 30 sec prolonged stimulation and 1 sec later, a second 500 ms pulse. The asterisk denotes a significant difference between the first and the second pulse of CO_2_ (N = 6, Wilcoxon matched-pairs test, p<0.05). **B.** The ab1C response to 0.5% or 10% CO_2_ was not affected by long-lasting stimulation with 5% vinegar. Only the responses to pre- and post-adaptation stimulation are shown. **C.** The ab1C response to the same stimuli was not affected when the pulse was applied during stimulation with 5% apple cider vinegar. **D.** Responses of ab1A to stimulation with ethyl acetate (0.001%) or 5% vinegar before and after stimulation with 0.5% CO_2_. In all cases, there was no significant difference between the first and the second stimulation (N = 4–6, Wilcoxon-pairs test, p>0.05). All values are means ± SEM.

Because CO_2_ is detected uniquely by ab1C neurons and vinegar strongly excites ab1A and to a lesser extent ab1B, the modulations of behaviour we observed may be due to interactions occurring at the level of the ab1 sensillum. This could be a direct effect of CO_2_ and odorants interacting at the level of a single receptor neuron, *i.e.* vinegar odour affects the response of ab1C neurons to CO_2_ (see Turner and Ray, 2009), and/or CO_2_ affects the ab1A/B response to vinegar. Alternatively, the effect could be indirect, such as signalling between the activated ab1C and ab1A/B neurons. We first investigated whether stimulating female ab1 sensilla with vinegar modified the sensitivity of ab1C neurons. Using a protocol similar to the one described by de Bruyne et al. [33], we compared the response of ab1C neurons to a pulse of CO_2_ shortly before and immediately after a long (30 seconds) stimulation. When using 0.5% CO_2_ for both short pulses as well as for the long stimulation, the ab1C neuron clearly adapted during the long stimulation of CO_2_, dropping its firing rate to about 35% of the initial response (Figure 4A). This adaptation significantly reduced the response to the post-adaptation pulse when compared to the response before adaptation (Wilcoxon matched-pair test, p<0.05). We then used vinegar as long stimulus and found no difference between the responses to the pulse of CO_2_ before or after vinegar (Figure 4B, Wilcoxon matched-pair test, p>0.05). Adaptation was observed in ab1A/B but not in ab1C (not shown). Next we moved the occurrence of the second pulse forward to overlap with the vinegar long stimulus, effectively mimicking a situation of a fly moving in a four-field olfactometer from a vinegar to a vinegar+CO_2_ field [15]. In this situation the vinegar stimulation still did not change the response of the ab1C neuron to CO_2_ (Figure 4C, Wilcoxon matched-pair test, p>0.05). Thus, ab1C sensitivity to CO_2_ was not altered by the activity of ab1A/B in response to vinegar. We also tested the reverse hypothesis, *i.e.* whether CO_2_ can modulate the responses of neighbouring ab1A/B neurons by stimulating the ab1 sensillum with pulses of ethyl acetate or vinegar before and after a long stimulation with CO_2_. Responses of ab1A/B neurons did not change after CO_2_ exposure (Figure 4D, Wilcoxon matched-pair test, p>0.05). These experiments show that the modulation of female behaviour is not due to direct nor indirect effects of CO_2_ and vinegar on ab1C and ab1A/B neurons.

### Coding of Temporal Properties of Pulsed Stimuli in CO_2_− and Vinegar-responsive Neurons

**Figure 5:**
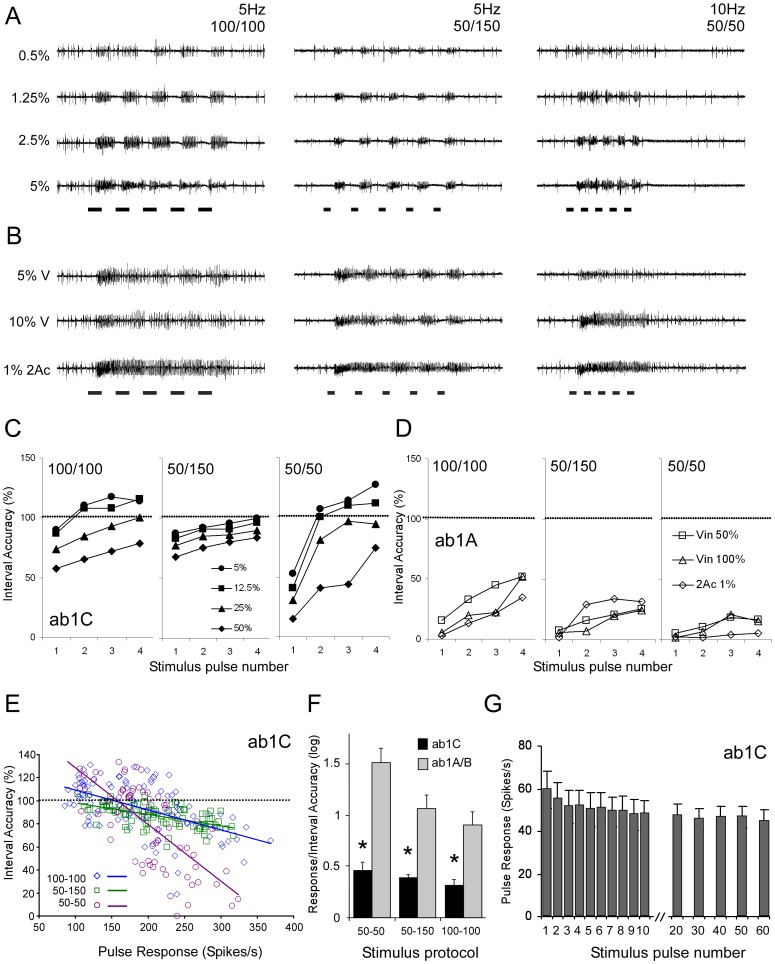
Female ab1C neurons encode pulsed stimulations more accurately than ab1A neurons. A. Recording traces showing the accuracy of responses from female ab1C neurons to a series of CO_2_ pulses at four different concentrations and three different stimulus protocols. Five 100 ms pulses were delivered at a frequency of 5 Hz or five 50 ms pulses at 5 Hz and 10 Hz. **B.** Recordings as in A showing responses from ab1A to stimulation with vinegar (V) at 5% and 10% dilution and ethyl acetate (2Ac) at 0.001%. **C**–**D.** Accuracy of pulse-interval encoding expressed as the longest inter-spike interval divided by the actual inter-pulse interval for ab1C (C) and ab1A (D) respectively. The accuracy is calculated for the interval following each successive stimulus pulse. N = 4–10 **E.** Negative correlation between interval accuracy and the ab1C neuron response to the preceding pulse. Different concentrations and successive pulses were pooled. **F.** The ratio between pulse response and interval accuracy is different for ab1C and ab1A. Asterisk denotes significance (Mann-Whitney U-test, p<0.05) **G.** Responses of ab1C neurons to repeated 500 ms stimulations with 0.5% CO_2_ at a frequency of 1 Hz showed a low level of adaptation. N = 7. Values are means ± SEM.

The physiological properties of *Drosophila* ORNs have mostly been measured by exposing them to standard short (0.5–2 seconds) pulses of odorants. However, flying insects traversing natural odour plumes often encounter rapid pulses of different intensities, and this is likely to be the case in our two-trap cage assay where the odour stimuli emerging from the funnels develop filamentous plumes (Figure S1). To investigate how accurately CO_2_- and vinegar-sensitive neurons encode rapidly fluctuating stimulations, we tested their responses to different concentrations of odorants in three different protocols of pulsed stimuli. Four doses of CO_2_ were tested with three patterns of stimulation: 100 ms stimulations at 5 Hz, 50 ms stimulations at 5 Hz, and 50 ms stimulation at 10 Hz (Figure 5A). We found that ab1C neurons were able to follow all three patterns of stimulation quite accurately, with better resolution at lower CO_2_ concentrations and lower pulse frequencies. We then compared this high efficiency of ab1C to that of ab1A/B when challenged with pulses of vinegar at two different doses and ethyl acetate using the same protocols (Figure 5B). These neurons encoded intermittent stimulation very poorly, displaying only weak reductions in spiking activity between odour pulses. We quantified how accurately the neurons encode the period between stimulations by measuring the longest inter-spike interval as a proportion of the actual interval between pulses (Figure 5C–D). The accuracy increased across the four sequential inter-pulse intervals: ab1C neurons reach 100% accuracy in most cases except the highest concentration at the 50/50 protocol (Figure 5C). By contrast, the ab1A/B neurons were never able to follow the pulses with more than 50% accuracy (Figure 5D). With ethyl acetate at 10 Hz (50/50) the neurons responded continuously as if the stimulation was not pulsed. For both ab1C and ab1A/B, interval accuracy appeared to depend on odour concentration. However, when the interval accuracy is plotted against the response to the previous pulse, accuracy is negatively correlated with response magnitude (Figure 5E). Therefore we pooled the data across concentrations and stimulus pulse number to compare the ratio of response magnitude to interval accuracy for ab1A/B and ab1C neurons (Figure 5F). The ratios were significantly different for all three protocols, indicating clear differences in the abilities of these neurons to encode rapidly changing stimulations.

We further investigated the capability of ab1C neurons to follow sequential stimulation by examining the responses to an extended period of repeated 500 ms stimulations. The responses weakly decreased over the first three stimulations but remained practically constant afterwards. Compared to the quick reduction of the response to prolonged continuous stimulation (Figure 4A), this result demonstrates that ab1C neurons adapt and disadapt relatively fast and are optimised for accurate responses to rapidly changing stimuli.

## Discussion

### Context-dependent Hedonic Responses to the Combination of CO_2_ and Vinegar

The role of olfactory stimuli in *Drosophila* orientation behaviour has been studied in a variety of assays [34]–[37], where, in addition to odour stimuli, flies experienced different conditions regarding e.g. light, space, airflows, starvation methods, choice or no choice. Not surprisingly, fly behaviours towards many odorants may be inconsistent across the different assays. However, hedonic values for CO_2_ and vinegar have been proven relatively robust [11]–[14]. In the four-field olfactometer we initially found that flies spent more time in a field supplied with vinegar odour but avoided entering a field with CO_2_ [15]. In this study, we confirmed these results in a two-trap cage assay. We were also able to confirm that combining the two stimuli induced a female-specific behavioural response. However, these responses differ between the two assays. In the four-field olfactometer the avoidance of CO_2_ was enhanced by the presence of vinegar in the background, while in the two-trap cage assay the presence of CO_2_ raised the attractiveness of vinegar odour.

We consider four factors that may contribute to the different behavioural outcomes in the two-trap cage assay and the olfactometer assay: stimulus presentation, the integration of spatial and olfactory stimuli, timing, and group effects. Firstly, the odour presentation was different in the two assays. Whereas odour concentrations were constant and homogeneous in the four-field olfactometer, the odours in the two-trap cage were diluted and broken up in the plumes above the flasks (Figure S1). Flies experienced varying concentrations of odorants and pockets of clean air while they flew through the two plumes. It is important to note that increased attraction only occurred at relatively low concentrations of CO_2_, the strongest effect was observed at 0.1%. In the four-field olfactometer females avoided concentrations as low as 0.02% CO_2_ [15]. Secondly, the spatial conditions were different between the assays. In the four-field olfactometer the flies’ mobility and active interaction with the stimulus is always limited and flies encountered the olfactory stimulus only by walking around in the arena. Furthermore, the flies studied in the olfactometer assay could not rely on other cues normally used in orientation behaviour such as optical flow and visual targets because experiments were conducted in the dark [15]. By contrast, in the cage assay behavioural choices were made in flight rather than while walking, and in the presence of clear visual targets. Thirdly, flies in the two-trap cage assay had ample time (5 hrs) to settle down and initiate a search at an appropriate time whereas in the four-field olfactometer they were released under more stressful circumstances, had only limited time (10 min) to orient and could not escape the situation by flying away. Attraction to potential resources may be less of an urgency and depend more on motivation [36]. The two-trap cage assay may present a more natural frame of reference for host searching behaviour including distance orientation, landing and local search on a patch [38]. Finally, because we tested groups of flies rather than individuals, some fly movements may be influenced by those of others as when males follow females. Such a group effect has been seen when the presence of “primer” flies enhances the responses of “follower” flies [39].

### Vinegar Components and Neuron Sensitivity

Apple cider vinegar activates several glomeruli in the *Drosophila* antennal lobe, and at least two of them, DM1 and VA2, were necessary for attraction in a four-field olfactometer [11]. These glomeruli receive inputs from neurons expressing Or42b and Or92a respectively, and our results confirm the expression of Or42b receptor in ab1A and Or92a in ab1B. We show here that their excitation by ethyl acetate and acetoin is comparable to that by vinegar. In addition, vinegar and ethyl acetate were found to also excite the ab2A neuron (DM4, [ref11]). Interestingly, the major components of vinegars, acetic acid and ethanol, did not activate any of the neurons we recorded from. These compounds have been shown to attract *Drosophila* [21], [40], [41]; hence, it is likely that they excite other ORNs. In addition, responses to ethyl acetate and acetoin were similar in males and females. Commercial vinegar has been shown to attract *Drosophila* flies in a range of studies but maybe a well defined blend of its components could be used in future studies.

### Sexually Dimorphic Behaviour but No Sexually Dimorphic Sensory Pathways

Both male and female flies feed on fermenting fruits and are attracted to vinegar. *Drosophila* flies aggregate on a food patch where mating and other social interactions take place [42]. However, we observed that females were attracted to a combination of CO_2_ and vinegar wheras males were not. The simplest explanation for the lack of male responses to the addition of CO_2_ would be that their sensory neurons are less sensitive to it. However, we did not find any differences in the response properties of male and female ab1C neurons. Even though males can perceive CO_2_ equally well, they do not respond behaviourally to it the same way as females do. Similarly, we did not observe differences in vinegar sensitive neurons of male and female flies. Therefore, the female-specific behaviours to the CO_2_-vinegar combination in both behavioural assays are not caused by modifications in neurons in ab1 sensilla. Consistent with this we did not see a difference in the behavioural response to the individual stimuli; vinegar and CO_2_ were equally attractive or repellent to males and females. We conclude that the female-specific oriented response to the CO_2_-vinegar combination cannot be explained by a difference in female response to CO_2_ or vinegar alone, but rather by an interaction at the level of ORNs or by an integration in the central nervous system.

### No Interaction between CO_2_ and Vinegar in the Periphery

Several lines of evidence suggest that olfactory information can be integrated at the level of ORNs [10], [43], [44]. Turner and Ray [9] showed that some odorants directly inhibit the ab1C neurons and reduce CO_2_ avoidance behaviour. In the present study we did not observe inhibition of ab1C neurons in presence of vinegar, nor a reduced avoidance, but rather an increased attraction. We also found that simultaneous stimulation with vinegar and CO_2_ did not induce any modulation of the response by vinegar-sensitive neurons. Our results thus indicate that the behavioural effect of the CO_2_-vinegar combination is not due to direct nor indirect interactions at the level of ab1 sensilla. This suggests that other mechanisms, most likely operating at the brain level, are responsible for the change in response to CO_2_ when perceived with vinegar odour. Oriented behaviours have been shown to be modulated by local interneurons in the antennal lobes [45], [46]. There is also evidence for sexually dimorphic neurons in the brain, although most functional evidence focuses on their role in sexual behaviours [47], [48].

### Special Properties of CO_2_-sensitive Neurons

It has been suggested that the special nature of the CO_2_ neural pathway is somewhat reminiscent of the pheromonal pathway [30]. Here we show that some physiological properties of ab1C neurons are remarkable. The dose-response relationship at the onset of stimulation suggests flies can accurately discriminate CO_2_ concentrations between 0.01 and 0.1% because firing rates increased from 10–70 spikes/s. In addition, adaptation to tonic stimulation was quick, and there was a rapid response to the offset of stimulation which was also dose-dependent. Disadaptation is equally rapid, leading to consistent responses to sequential stimulations. As a result *Drosophila* ab1C neurons are particularly good at detecting rapid variations in CO_2_ concentrations around ambient levels. Their detection threshold is comparable to CO_2_-sensitive neurons of mosquitoes which also show similar sensitivity to on- and offset of stimulation [49], [50]. *Drosophila* ab1C neurons do not monitor constant background CO_2_ levels (Figure 3B, 3D) allowing them to respond to variations in concentration that are smaller than the background. The ranges of background concentrations we tested are well within levels occurring in natural microhabitats [51]. Although adaptation was rapid, high concentrations of CO_2_ did not induce complete adaptation (Figure 4A), and flies would still be able to perceive continuous stimulation. This means that ab1C neurons would be able to constantly monitor CO_2_ well above ambient concentrations during walks inside the odour fields of a four-field olfactometer or while entering the funnels of the two trap cage used in this study. It would be interesting to see whether the constant sensitivity in low-level backgrounds and rapid adaptation/disadaptation to high levels we observe here use the same molecular mechanisms.

The rapid on- and offset properties of ab1C neurons enable them to encode CO_2_ pulses much more accurately than ab1A/B neurons. Considering the speed and manoeuvrability of flies, fast encoding of on-off events is crucial for in-flight orientation to odour emitting objects [13], [52]. However, most ORNs are optimised to detect a variety of odorants and do so with varying temporal firing properties [10], [24], [53]. Even though post-stimulus quiescence is observed in some ORNs in response to certain odorants, most encode odour pulses less accurately than CO_2_-sensitive neurons. Our results demonstrate that ab1C neurons are much better at following CO_2_ pulses than ab1A/B neurons are at following pulses of vinegar. The latter may provide important information about the nature of the odour source but may not accurately represent the temporal properties of the stimulus.

### Complex Roles of CO_2_ in Fly Behavioural Ecology

Because CO_2_ is such a ubiquitous stimulus, varying only in concentration arising from different sources, it may not be suitable to consider CO_2_ a specific odour cue, as it is for instance with sex-pheromones. Flies invariably encounter changes in CO_2_ levels combined with other odorants. For example, the quantities of CO_2_ produced by fruit vary with ripening stages [15] and flies are more attracted to over-ripe than unripe fruit [36]. The CO_2_ levels on over-ripe fruit may be increased by the fermentation from active yeast, which *Drosophila* prefers over killed yeast [22]. Dissolved CO_2_, detected by gustatory neurons on the proboscis, has a positive hedonic value, increasing acceptance of food sources [54]. Therefore, the combination of particular blends of odorants and levels of CO_2_ may enable the flies to distinguish ripening stages of fruit as well as the presence of favourable (yeast) and unfavourable microorganisms. These evaluations will depend on behavioural context. For instance, when a *Drosophila* is flying toward distant patches of fruit, the detection of CO_2_ may raise the attractive value of a food-related odour. A similar “sensitising” role for CO_2_ to an attractive stimulus has been observed in mosquitoes [18]. However, after landing on a fruit, a fly may perform local searches, avoiding increases in CO_2_, which in this situation take on a negative value and are indicative of adverse conditions such as stressed flies [14], predators or unsuitable levels of microorganisms. This context-dependent effect was also observed in moths, where CO_2_ acts as an orientation cue toward distant scented flowers but has no effect on local feeding behaviour [55]. The hedonic value of the CO_2_-vinegar combination will also depend on sex, as proven by the female-specific behaviour to that combination. This specificity therefore suggests a role in oviposition behaviour. Males may not rely on the fruit ripening level for their food source finding, but rather on the presence of conspecific flies (our observations; [56]). In addition males and females have different nutritional needs. Males need less energy and females in particular need yeast for egg-development [57].

In flight, the superior temporal accuracy of ab1C neurons may also improve the spatio-temporal resolution of internal representations of stimulus distribution by “sharpening” the borders of odour filaments in time. The fact that CO_2_-sensitive neurons project only ipsilaterally to their glomeruli, whereas all other ORN classes have both ipsilateral and contralateral projections [29], suggests that CO_2_ stimuli are encoded in a different way in the brain. Whereas the identity of odorants in a mixture may have a positive hedonic value, the addition of CO_2_ might improve the precision with which these odours can be tracked. Even though *Drosophila* does not require pulsed stimulation for upwind flight as some moth species do [35], CO_2_ at low concentrations may enhance attraction by improving navigation through turbulent odour plumes.

In conclusion, our results indicate that the hedonic values of odour stimuli depend on behavioural context and that it is misleading to label single odorants as either attractant or repellent. Although we do not as yet understand the underlying neural mechanisms, our data show that the orientation of *Drosophila* to an odour blend depends on the precise concentrations of its components, the sex of the fly and the behavioural context in which the mixture is presented.

## Materials and Methods

### Insects

*Drosophila melanogaster* (Meigen) flies were reared on standard yeasted cornmeal-syrup medium at 25°C, 50–60% relative humidity, and 12 h:12 h L:D photoperiod. Wild-type flies were Canton S while transgenic lines were *w;P[Or42b-Gal4]^141t2.1^/CYO;* and *w;+;P[Or92a-Gal4]^131t1.4^/TM3* (kindly provided by B. Dickson), and *w;+;P[UAS-DTI]* (kindly provided by A. Thum). DTI is a Diphtheria toxin that mediates cellular apoptosis. Flies were 5–10 day old on the day of the experiment, except for the transgenic ones that were at least 3 week old.

### Single-sensillum Recordings

The extracellular recording procedure was largely as described previously [24]. A single fly was inserted into the end of a truncated plastic pipette tip, and the right antenna was held by a glass micropipette on a glass coverslip in order to access the basiconic sensilla. The antenna was visualised under a microscope with a 1000× magnification (Olympus, BX50WI). Electrical activity was recorded by inserting a glass electrode into a sensillum and the reference electrode into the left eye. Electrodes were filled with a saline solution (15 mM KCl). Changes in trans-epithelial voltages originating in electrical activity from the ORNs of one sensillum were transmitted to an Ag/AgCl wire linked to a high impedance 10× pre-amplifier (Syntech, Hilversum, The Netherlands). Signals were then digitally amplified 100× to a total of 1000× by a device interface (IDAC: Synthech, Hilversum, The Netherlands) and sent to a computer equipped with Autospike software (Syntech, Hilversum, The Netherlands). High pass (AC) and unfiltered (DC) signals were recorded simultaneously for 8 s to capture action potentials (spikes) and slower sensillum potentials respectively. This included a 2 s pre-stimulation period and a 500 ms stimulation period.

ORN responses were quantified by counting the number of spikes during these two periods divided by the time to obtain the firing rate in spikes per second. Pre-stimulus activity was then subtracted from the response during stimulation to get an increase (or decrease) in the spike frequency relative to the pre-stimulus frequency. Recordings were restricted to large basiconic sensilla (ab1, ab2, and ab3) and to no more than two sensilla from the same class per fly. We analysed only recordings where spikes from ab1C neurons could be clearly separated from others.

Differences between males and females in response to the same stimulus were tested using Mann-Whitney U-test. A Wilcoxon matched pairs test was used to compare responses to the first and second stimulations during cross-stimulation tests. All statistics were done using Statistica software. For analysis of responses to pulsed stimuli we counted spikes during each pulse and calculated the rate in spikes per second. We also measured the longest inter-spike interval (ms) in the period between odour pulses to determine the interval between two subsequent bursts. The accuracy of encoding the temporal structure of pulsed stimuli was calculated as the “interval accuracy”, the ratio of the duration between two responses to the actual interval between the odour pulses.

### Odour Stimulations

The antenna was supplied with humidified synthetic air (i.e. 0% CO_2_) at 1200 ml/min from a glass tube. A second airflow of 120 ml/min was added by inserting the needle of a 5 ml empty polypropylene syringe into a hole in the glass tube 5 cm upstream of the preparation. The hole also received the needle of the syringe containing an odour stimulus. During stimulation, air was switched from the empty to the stimulus-syringe by a solenoid valve triggered by a stimulus delivery controller (Syntech, Hilversum, The Netherlands). Carbon dioxide was taken with a 5 ml syringe from gas bottles (Air Liquide, Germany) at 5% in synthetic air or 100%. Step dilutions were done by diluting with appropriate quantities of synthetic air. Odorant solutions (10 µl) were loaded on filter paper inserted in a 5 ml syringe. Organic apple cider vinegar purchased from a commercial supplier (Bio-Zentrale, Stubenberg, Germany) was used pure or diluted in distilled water. Synthetic odorants were tested at the highest purity available: Acetic acid (Fluka 45727), >99.0%, ethanol (Sigma-Aldrich 459844), >99.5%, ethyl acetate (Fluka 58958), >99.9%, acetoin (Fluka 0540), >97%. We used these volatiles as they occur in apple cider vinegar [32] diluted 50% in distilled water. The dilutions (v/v) were: acetoin and ethyl acetate at 0.01%, ethanol at 0.1%, and acetic acid at 3% (Figure 2). Ethyl acetate was diluted in paraffin oil for experiments shown in Figures 4 & 5. Adding the airflow from the stimulus syringe to the main airflow led to a further 10% dilution before reaching the antenna. Therefore all concentrations are given as those reaching the flies, i.e. ten times less than those applied to the syringe. To increase the background level of CO_2_ in the main airflow, we either replaced synthetic air with pressurised air containing 0.03% CO_2_ (Air Liquide, Germany) or we added 18 ml/min of 5% CO_2_ in synthetic air (Air Liquide, Germany) to the main CO_2_-free airflow resulting in a 0.07% CO_2_ level. All gas flows were measured with precision rotameters.

To test for a possible effect of apple cider vinegar on the response to CO_2_, a dynamic equilibrium was established in a 50 ml glass flask by blowing air at 120 ml/min over 20 µl of 50% v/v vinegar applied on a filter paper inserted. A solenoid valve directed the flow either to a 5 ml syringe whose needle was inserted in the glass tube hole to stimulate the antenna or to the exhaust system. Filter papers were used for a maximum of 10 minutes. The antenna was first stimulated with a 500 ms pulse of CO_2_ 15 seconds before being stimulated with vinegar for 30 seconds then stimulated a second time with a 500 ms pulse of CO_2_ either 5 seconds before the end of stimulation with vinegar, or 1 second after. Similarly, to test the effect of CO_2_ exposure to the responses to vinegar and ethyl acetate, a 120 ml/min flow of 5% CO_2_ was continuously blown from a gas bottle and directed by a solenoid either to the main airflow during stimulation, or to the exhaust system.

For studying ORN responses to pulsed stimuli we used three stimulation protocols, all consisting of 5 pulses: 100 ms stimulation followed by 100 ms clean air (i.e. 5 Hz frequency); 50 ms followed by 150 ms clean air (5 Hz); and 50 ms followed by 50 ms clean air (10 Hz). For simplicity, we refer to these as 100/100, 50/150, and 50/50 patterns.

### Behavioural Assays

#### Two-trap cage assay

Odour preferences were tested in a plastic framed cage (30×30×30 cm) covered with polyamide mesh except on the front side, which had a sliding Plexiglas panel (Figure 1A). Two plastic funnels (6.5 cm diam.) were held on the bottom of the cage by a truncated pipette tip inserted in a silicon plug that fit a 250 ml glass flask placed below the cage. These traps were designed so that flies could not escape once in the flask. Air entered the flasks through another pipette tip in the silicon plug. Pumped room air was humidified and split into two streams each set at 240 ml/min. Air was carried by 5 mm diam. tubing that entered the cage through foam plugs in the front panel. A wet sponge cloth on the base of the cage supplied humidity.

CO_2_ from gas bottles (5% in synthetic air or 100% CO_2_; Air Liquide, Germany) could be added to one airstream and mixed to final concentrations of 0.02%, 0.1% or 1% above ambient level by adjusting the flow with a precision rotameter. Pressurised air (Air Liquide, Germany) was used as a control instead of CO_2_ and stimuli were interchanged to avoid positional effects. Flasks could also contain either 3 ml of apple cider vinegar or distilled water. A 7 watt light bulb below the cage provided the only dim light source, allowing the flies to see the funnels while preventing strong visual bias.

Flies were cold anaesthetised, and about 50 males and 50 females were selected and starved overnight on agar. The vial containing the flies (n = 93–116) was introduced in the centre of the cage (through a nylon stocking in the front panel, not shown in the figure) and flies were allowed to passively disperse. The experiment ran for 5 hours (14∶00–19∶00) at room temperature (20 to 25°C) after which the flies trapped in each flask were counted, and the percentage in each flask recorded. Glass flasks and funnels were cleaned with alcohol and distilled water after each test.

For each trap combination, the experiment was replicated 9–10 times. The numbers of flies in the test trap, control trap and those remaining in the cage were compared with Friedman-ANOVA followed by Wilcoxon signed rank tests with sequential Bonferroni correction (p<0.05). When comparing the overall attraction to both vials (Figure 1B) across various conditions, differences between numbers of trapped flies were assessed by a Kruskal-Wallis test followed by Mann-Whitney-U tests with Bonferroni correction (p<0.05). Statistics were performed with the Statistica software.

## Supporting Information

Figure S1 **Odour plume structure in the two-trap cage assay.** The airstream from one of the funnels was visualised using cigarette smoke. Note the broken up filamentous structure in the air above the funnel.
(TIF)

Figure S2 **Comparable levels of CO_2_ avoidance using three different behavioural assays: T-maze, four-field olfactometer and two-trap cage.** A concentration of 1% CO_2_ was tested against air in the two-trap cage assay as described (data from Figure 1C). The T-maze [34], [58] consisted of a central sliding compartment (2 cm diam., 11% of the total internal volume) in which groups of 30–50 flies (males and females) were loaded and moved down so that they could choose between the two arms during 3 minutes. CO_2_ was alternatively added to one arm to minimise any orientation bias other than related to the odour stimulus. An avoidance index was calculated to quantify responses in the two assays where AI = (number of flies in control – number of flies in test)/total number of flies in both. An index of 1 indicates that all of the trapped flies were in the control flask or arm (avoidance), whereas an index of 0 indicates an equal number of flies in both flasks. For the four-field olfactometer data was taken from Faucher et al [15], and an avoidance index was calculated as follows: AI = 2× (percentage time in test field)^0.5^−1 as in [11].
(TIF)

Figure S3 **Ab1C neurons respond to concentration decrease of CO_2_.** Responses from ab1C neurons to 500 ms pulses of decreased CO_2_ concentrations in a 0.07% CO_2_ airstream. N = 13–18, values are means ± SEM.
(TIF)
