# Supplementary figures and images for: Interactions of Carbon Dioxide and Food Odours in Drosophila: Olfactory Hedonics and Sensory Neuron Properties
Source: PLoS One. 2013 Feb 15;8(2):e56361. doi: 10.1371/journal.pone.0056361 (PMC3574157; doi:10.1371/journal.pone.0056361)

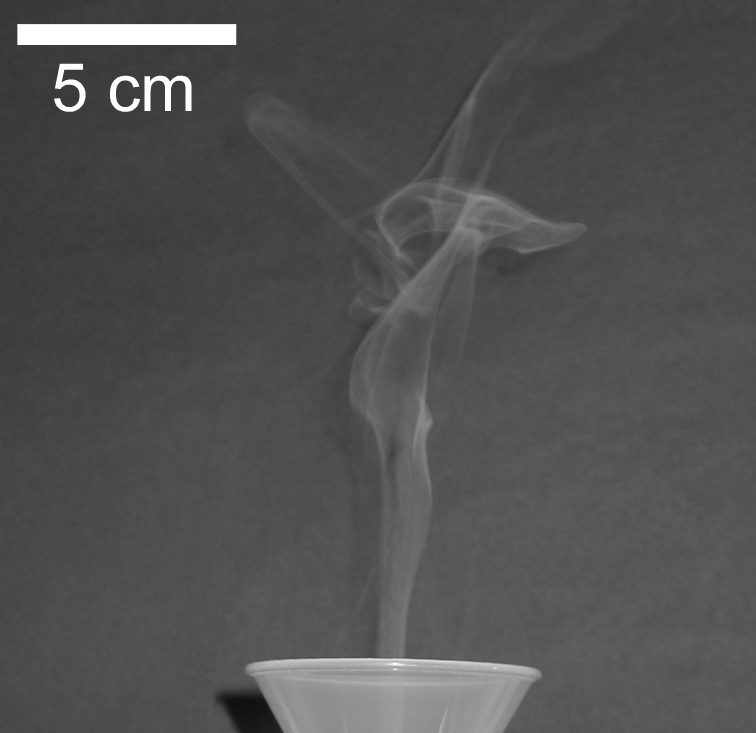

Supplement: Figure S1 — Odour plume structure in the two-trap cage assay. The airstream from one of the funnels was visualised using cigarette smoke. Note the broken up filamentous structure in the air above the funnel. (TIF) [file pone.0056361.s001.tif]

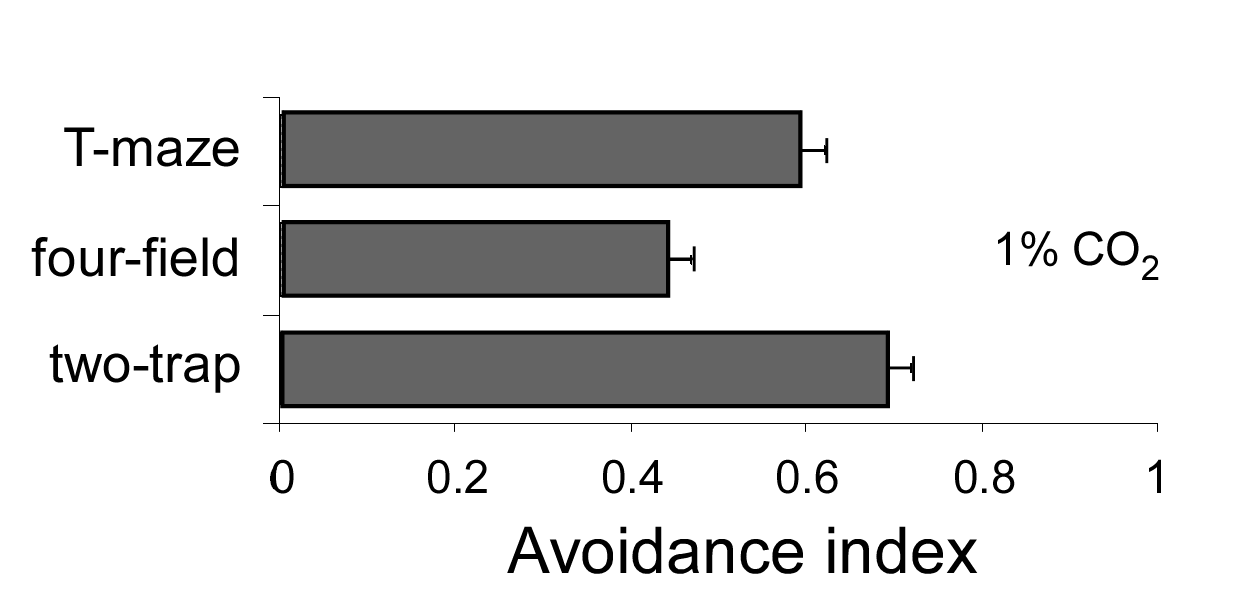

Supplement: Figure S2 — Comparable levels of CO2 avoidance using three different behavioural assays: T-maze, four-field olfactometer and two-trap cage. A concentration of 1% CO2 was tested against air in the two-trap cage assay as described (data from Figure 1C). The T-maze [34], [58] consisted of a central sliding compartment (2 cm diam., 11% of the total internal volume) in which groups of 30–50 flies (males and females) were loaded and moved down so that they could choose between the two arms during 3 minutes. CO2 was alternatively added to one arm to minimise any orientation bias other than related to the odour stimulus. An avoidance index was calculated to quantify responses in the two assays where AI = (number of flies in control – number of flies in test)/total number of flies in both. An index of 1 indicates that all of the trapped flies were in the control flask or arm (avoidance), whereas an index of 0 indicates an equal number of flies in both flasks. For the four-field olfactometer data was taken from Faucher et al [15], and an avoidance index was calculated as follows: AI = 2× (percentage time in test field)0.5−1 as in [11]. (TIF) [file pone.0056361.s002.tif]

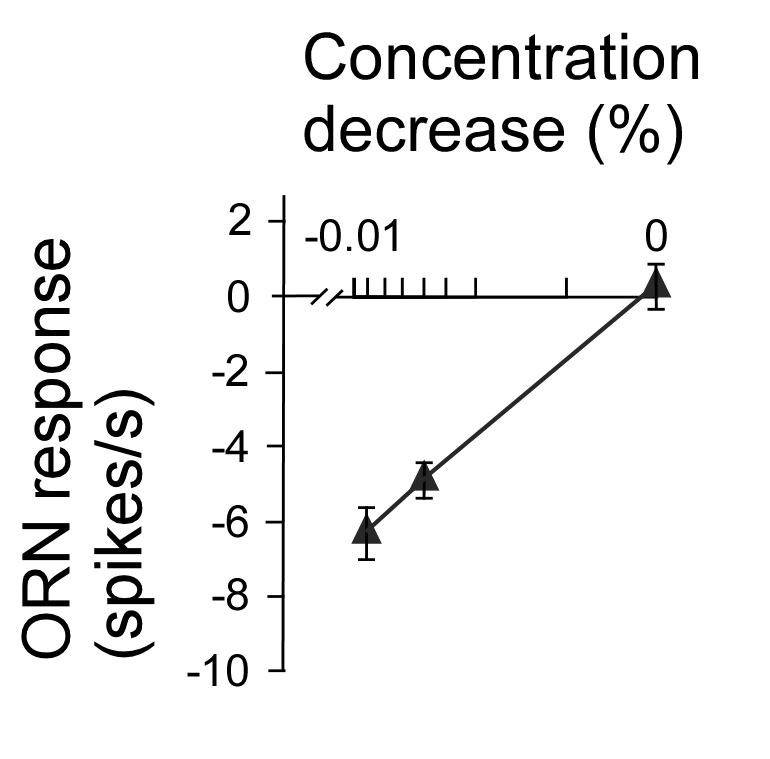

Supplement: Figure S3 — Ab1C neurons respond to concentration decrease of CO2. Responses from ab1C neurons to 500 ms pulses of decreased CO2 concentrations in a 0.07% CO2 airstream. N = 13–18, values are means ± SEM. (TIF) [file pone.0056361.s003.tif]
